# Lipid Profile in Pre-dialysis and Post-dialysis End Stage Renal Disease Patients: A Cross-Sectional Comparative Study in Lucknow, India

**DOI:** 10.7759/cureus.18240

**Published:** 2021-09-24

**Authors:** Manpreet Saini, Amrita Vamne, Vijay Kumar, M S Chandel

**Affiliations:** 1 Department of Biochemistry, Index Medical College and Research Centre, Malwanchal University, Indore, IND; 2 Department of Community Medicine, Mayo Institute of Medical Sciences, Barabanki, IND; 3 Academics, Index Medical College and Research Centre, Malwanchal University, Indore, IND

**Keywords:** hemodialysis, dyslipidemia, lipid profile, end stage renal disease, chronic kidney disease

## Abstract

Background: Chronic kidney disease (CKD) patients are at elevated risk of cardiovascular diseases (CVD) due to altered lipid profiles. Dyslipidemia is maximal in end stage renal disease (ESRD) patients and there is insufficient data on the impact of hemodialysis on lipid profile. The present study was aimed to evaluate the effect of hemodialysis on lipid profile of CKD patients.

Materials and Methods: This cross-sectional study was conducted on 50 CKD patients on hemodialysis from three randomly selected hospitals of Lucknow, Uttar Pradesh, India, between March - May 2021. Serum lipid profile was analysed before and after hemodialysis session by using an auto analyzer. The mean values of different lipid parameters before and after hemodialysis were calculated and the difference between them was analyzed by using paired t-test. A p-value of <0.05 was considered to be statistically significant.

Results: In this study, very low density lipoprotein (VLDL) levels decreased significantly after hemodialysis. Total cholesterol (TC), low density lipoprotein (LDL) and triglyceride (TG) levels were also significantly lowered. High density lipoprotein (HDL) was the only lipoprotein that increased after dialysis although this increase was non-significant.

Conclusion: Adequate dialysis and time bound monitoring of various components of lipid profile can help CKD patients by decreasing risks for cardiovascular complications.

## Introduction

Chronic kidney disease (CKD) is abnormalities of kidney structure or function, present for more than three months with implications for health and a progressive decline in glomerular filtration rate (GFR) [1]. CKD is a global health issue with increasing incidence and prevalence every year. The exact prevalence may be even more as CKD in early stages is mostly asymptomatic. CKD has five stages and the last stage which demands renal replacement therapy either in the form of' dialysis or renal transplantation for its management is called end stage renal disease (ESRD) and over the last 10 years the incidence and prevalence of ESRD has doubled [2].

Many studies have found that more than 50% of CKD patients rather than progressing towards ESRD, die early from cardiovascular diseases (CVD) [3,4] and the dyslipidemia is the underlined cause of developing CVD [5]. The severity of dyslipidemia parallels the gradual decrease in renal function and this disturbed pattern of lipoprotein metabolism is seen even in the early stages of chronic renal failure (CRF) [6]. Dyslipidemia seen in CKD patients is highly atherogenic and is associated with development of atherosclerotic cardiovascular disease and it is maximal in patients with ESRD. Dyslipidemia can even independently act as a predisposing factor for the progression of renal damage. The detrimental effect of dyslipidemia on the progression of CKD underlines a number of evidences including impaired blood supply due to atherosclerosis which further damages the kidneys [7]. It is a well known fact that the treatment of dyslipidemia plays a vital role in the prevention of CVD in the general population but its treatment in dialysis patients is still inconclusive due to lack of evidence [8]. Moreover there is limited research regarding the effect of single renal dialysis (RD) session on lipid profile in ESRD patients. So, it is rationale to conduct a study on ESRD patients to see the impact of hemodialysis on lipid profile.

Our objectives were to evaluate the effect of hemodialysis on lipid profiles of CKD patients.

## Materials and methods

A hospital-based, cross-sectional, observational study was done in different hospitals of Lucknow, Uttar Pradesh, India from March 2021 to May 2021. The study protocol was approved by the Malwanchal University Ethics Committee, Indore, Madhya Pradesh, India (MU/Research/EC/Ph.D/2020/04) and informed consent was obtained from all the patients.

Based on available hospital records it was estimated that around 50-60 patients could be included in this study owing to restrictions of COVID-19 and available logistics with the authors. A total of three randomly selected hospitals located in different parts of Lucknow, Uttar Pradesh, were enrolled for the study and based on consecutive sampling, 20 patients irrespective of age and gender from each hospital were included. We had a total of 50 patients (29 male and 21 female) with CKD on maintenance hemodialysis after applying exclusion criteria. Patients with liver disease, diabetes mellitus, history of alcohol and smoking, obesity (body mass index ≥25 kg/m2), patients on a specific prescribed diet, hypolipidemic therapy and lifestyle modifications were excluded from the study as these factors could affect lipid profiles and alter our study findings.

Patients were hemodialyzed for at least three to four hours per session. A blood sample (5 ml) was collected from all patients before and after hemodialysis in a plain bulb taking all aseptic precautions. After collection, samples were centrifuged and serum was analysed for lipid profile. Serum total cholesterol (TC), triglyceride (TG), high density lipoprotein (HDL), low density lipoprotein (LDL) and very low density lipoprotein (VLDL) levels were analyzed by HumaStar 200 auto analyzer using an enzymatic colorimetric method.

Statistical analysis

Data were entered in Microsoft Excel after generation of a proper template. Data entered was then imported into Statistical Package for Social Science (SPSS) software version 16 (IBM Corp., Armonk, NY, USA) and analysis was done. Different parameters of lipid profile were denoted in terms of mean±SD and the paired t-test was used to analyze the difference in the mean values of lipid analytes before and after hemodialysis. A p-value of <0.05 was considered to be statistically significant.

## Results

A total of 50 CKD patients on dialysis were included in the study. Table 1 shows age and sex of the study subjects. Their age varied from 28 years to 76 years with the mean age of 49.17 ± 11.45 years. Out of 50 study participants, 29 were males and 21 were females. CKD patients needing dialysis were more common in the age group of 40-60 years. Association of age and gender with ESRD was not found to be statistically significant.

**Table 1:** Age and sex distribution of End Stage Renal Disease Patients

Age in years	Male	Female	Total	chi-square value	p-value
20-40	6	5	11	0.075	0.963
41-60	17	12	29		
≥60	6	4	10		
Total	29	21	50		

TC, TG, HDL, LDL and VLDL values in patients of ESRD before and after dialysis are depicted in Table 2. The levels of TC, TG, LDL and VLDL decreased after dialysis while HDL levels were increased. To compare the pattern of lipid profile between both the groups, p-value was calculated. The p-value <0.05 was considered significant. As can be seen, mean concentration of TC, TG and LDL was 178.98 ± 26.21 mg/dl, 111.54 ± 14.46 mg/dl and 129.25 ± 18.47 mg/dl respectively before dialysis and after dialysis it decreased to 166.18 ± 20.03 mg/dl, 106.08 ± 10.88 mg/dl and 118.84 ± 16.15 mg/dl respectively. This decrease was statistically significant (P = 0.007).

**Table 2:** Comparison of pattern of Lipid profile in Pre-dialysis and Post- dialysis End Stage Renal Disease Patients

Parameters Serum lipids (mg/dl)	Pre-Hemodialysis (N=50) (Mean ± SD)	Post-Hemodialysis (N=50) (Mean ± SD)	p-value (t-test)
Total Cholesterol	178.98 ± 26.21	166.18 ± 20.03	0.007
Triglyceride	111.54 ± 14.46	106.08 ± 10.88	0.035
High Density Lipoprotein	42.62 ± 7.43	45.46 ± 9.12	0.091
Low Density Lipoprotein	129.25 ± 18.47	118.84 ± 16.15	0.003
Very low density lipoproteins	34.03 ± 3.74	31.95 ± 2.63	0.001

In this study, VLDL levels also decreased significantly (P = 0.001) after dialysis. Mean concentration of VLDL before dialysis was 34.03 ± 3.74 mg/dl and after dialysis it decreased to 31.95 ± 2.63 mg/dl. The only lipoprotein which increased after dialysis was HDL. Mean HDL level was 42.62 ± 7.43 mg/dl before dialysis and 45.46 ± 9.12 mg/dl after dialysis, although this increase was not significant.

## Discussion

Decreased kidney function is being identified as an important risk factor for cardiovascular events and hospitalization due to high prevalence of dyslipidemia which increases the risk of death among CKD patients [9]. The risk of death is even more if the disease progresses further to ESRD. Rising prevalence of ESRD has put health care facilities around the world under enormous strain.

In our study, hypertension, long-term blockage of urinary tract and other kidney disorders like glomerulonephritis were the main factors leading to CKD as other causes which could alter the lipid profile of the patients were excluded. The present study comprised of CKD patients on dialysis between the age group of 28 to 76 years with the mean age of 49.17 ± 11.45 years. Maximum patients were in the age group of 40-60 years. A similar finding was reported from a study done by Latha and Varma in Hyderabad, India, where the mean age was 48.27 ± 10.24 years [10]. This may be reflective of the fact that ESRD affects the economically productive age group. Though this is in contrast with the study done in the Western population where the mean age was found to be >65 years. The reason might be the difference in incidence of diabetes and hypertension (common risk factors for CKD) along with other factors like genetic and socio-cultural factors, therapeutic process and the disease pattern causing CKD [11].

In our study, the mean level of TC was found to decrease after dialysis when compared to before dialysis. Mean concentration of TC in ESRD patients during pre and post-hemodialysis sessions was 178.98 ± 26.21 mg/dl and 166.18 ± 20.03 mg/dl respectively and this decrease was statistically significant (P = 0.007). The finding in our study is in accordance with the study done by Nagane and Ganu where they found that post-dialysis sample mean of TC was significantly low when compared to pre-dialysis sample mean [12]. Contrary to this, another study done by Baldi et al. showed modest but statistically significant increments in total cholesterol after a single hemodialysis session [13]. The current study also revealed that level of triglyceride was significantly decreased after dialysis when compared with pre-dialysis value. This observation is similar to the findings in the study of Nagane and Ganu [12] while in contrast to the findings by Tsitamidou et al. [14].

This study depicts that the levels of LDL and VLDL both were decreased after dialysis. Statistical findings of decrease in LDL and VLDL levels were significant. The results are in accordance with the study done by Nagane and Ganu [12]. In their study they found that post-dialysis mean concentration of LDL lowered significantly compared to pre-dialysis value while Zulbeari et al. did not find any significant change in VLDL level after dialysis [15]. A similar study done by Seres et al. reported an opposite finding where there was a rise in LDL level after dialysis. Mean concentration of LDL before and after dialysis was 94.81 ± 33.05 mg/dl and 123.2 ± 46.08 mg/dl respectively and this change was statistically significant [16].

In the current study, mean HDL value was higher after dialysis although the increase was not significant. Seres et al. also found an increase in HDL after dialysis which was statistically significant [16]. Nagane and Ganu also found that mean value of HDL was significantly increased in post-hemodialytic sample when compared with pre-hemodialytic sample [12].

The findings of this study were closest to Nagane and Ganu in contrast to other similar studies. The only limitation of our study was smaller sample size due to restrictions of COVID-19.

## Conclusions

In our study, we observed that end stage renal disease patients before dialysis have more risk of cardiovascular diseases and after dialysis there is an improvement in dyslipidemia. So we conclude that for further improving the life of chronic kidney disease patients in terms of non-development of risk factors for CVDs, adequate dialysis treatment and timely monitoring of lipid profile should be done along with other modes of therapy like proper advised diet, lifestyle modifications and lipid lowering treatment.
